# Imaging-based clinical decision tree enables risk stratification of extraprostatic extension before radical prostatectomy in prostate cancer patients

**DOI:** 10.1186/s13244-026-02305-5

**Published:** 2026-05-14

**Authors:** Charlie A. Hamm, Tim Rüterhenke, Georg L. Baumgärtner, Catherina Mayerosch, Simon Schallenberg, Maximilian Lindholz, Nick L. Beetz, Patrick Asbach, Tobias Penzkofer

**Affiliations:** 1 Department of Radiology, Charité—Universitätsmedizin Berlin, Corporate Member of Freie Universität Berlin and Humboldt-Universität zu Berlin, Berlin, Germany; 2 Berlin Institute of Health (BIH), Berlin, Germany; 3 Department of Pathology, Charité—Universitätsmedizin Berlin, Corporate Member of Freie Universität Berlin and Humboldt-Universität zu Berlin, Berlin, Germany; 4 Department of Nuclear Medicine, University Hospital Zurich, University of Zurich, Zurich, Switzerland

**Keywords:** Prostatic neoplasms, Magnetic resonance imaging, Prostate-specific antigen, Clinical decision-making, Surgery

## Abstract

**Objectives:**

Extraprostatic extension (EPE) significantly impacts surgical planning for prostate cancer (PCa) patients, influencing nerve-sparing surgery and neoadjuvant therapy decisions. However, Likert-scale-based radiological (r)EPE assessment lacks sufficient diagnostic accuracy for reliable clinical decision-making. Therefore, the aim was to evaluate rEPE scoring alongside clinical parameters to develop a clinically feasible decision tree for preoperative risk stratification.

**Methods:**

This retrospective single-center study included 429 consecutive PCa patients undergoing radical prostatectomy between January 2012 and October 2018. All patients underwent multiparametric MRI with PI-RADS scoring and rEPE grading (grades 0–3). Clinical parameters included PSA density (PSAD) and ISUP grade group (GG) at biopsy. Univariate and multivariate logistic regression identified predictors of EPE. A clinical decision tree was developed using binary classification to stratify patients into risk groups.

**Results:**

EPE was confirmed in 145 patients (33.8%). Multivariate analysis identified rEPE grade (OR 2.64, *p* < 0.001) and GG at biopsy (OR 1.41, *p* < 0.001) as independent predictors. The decision tree assigned 48% of patients to the high-risk (rEPE grade 3: 89% EPE risk) and low-risk group (rEPE < 3 + PSAD < 0.2 ng/mL² + GG < 4: 13% EPE risk), while 52% showed intermediate risk (28–45% EPE risk).

**Conclusions:**

The developed decision tree combining MRI-derived rEPE grading, PSAD, and biopsy GG enables reliable identification of patients at high and low risk for EPE. This tool supports informed decision-making regarding nerve-sparing surgery and neoadjuvant therapy, potentially contributing to personalized treatment planning.

**Critical relevance statement:**

Decision tree combining routine MRI-based and clinical markers reliably stratifies prostate cancer patients into high-risk and low-risk groups for EPE, supporting personalized surgical planning.

**Key Points:**

- EPE affects surgical planning decisions in prostate cancer patients.
- Combining EPE grade at MRI, PSAD, and biopsy grade improves risk stratification.
- The developed decision tree reliably stratified every second patient into distinct EPE-risk groups, potentially improving personalized surgical planning.

**Graphical Abstract:**

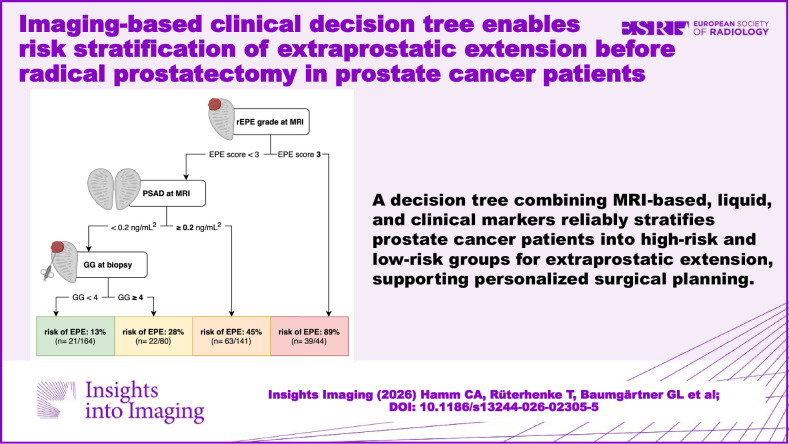

## Introduction

Prostate cancer (PCa) is the second most common cancer among men worldwide, with incidence expected to double by 2040 [1]. Early and accurate diagnosis is crucial for effective management and therapy. Prostate MRI has become essential in PCa diagnostics, particularly with the development of the Prostate Imaging-Reporting and Data System (PI-RADS) for biopsy decision-making [2]. While PI-RADS primarily aids in standardized imaging and risk-stratification of clinically significant PCa, MRI is also relevant for local staging, especially in predicting extraprostatic extension (EPE) [3–7]. EPE, where the tumor extends beyond the “prostatic capsule”, indicates a clinically significant cancer and thus accurate assessment of potential EPE is crucial for selecting appropriate therapy [8, 9]. Treatment decisions may require choosing between different approaches, including surgery vs radiation therapy or adding androgen deprivation therapy to radiation therapy [10–13]. Furthermore, nerve-sparing surgery is typically avoided in patients with high EPE risk due to the increased risk of positive surgical margins, which are associated with biochemical recurrence and metastasis [8, 11–14].

In this context, EPE scoring systems have been developed for more comprehensive and structured MRI reporting beyond PI-RADS [3–7]. Accurate radiological (r)EPE assessment potentially guides informed decision-making for surgeons and patients, particularly regarding the necessity of non-nerve-sparing operations. However, in the past, rEPE assessment lacked diagnostic accuracy to guide those decisions reliably and thus has not been translated into routine clinical decision-making [15].

Therefore, we aimed to evaluate the predictive ability of the rEPE scoring at MRI alongside other radiological and clinical parameters for EPE at prostatectomy. Moreover, we aimed to develop a clinically feasible decision tree to improve the assessment of the likelihood of EPE before surgery, thereby optimizing surgical planning and improving patient outcomes.

## Methods

This retrospective study complies with the Standards for Reporting of Diagnostic Accuracy guidelines and was approved by the institutional review board (EA1/271/16 und EA2/033/18) with waiver of written informed consent.

### Study sample

**Fig. 1:**
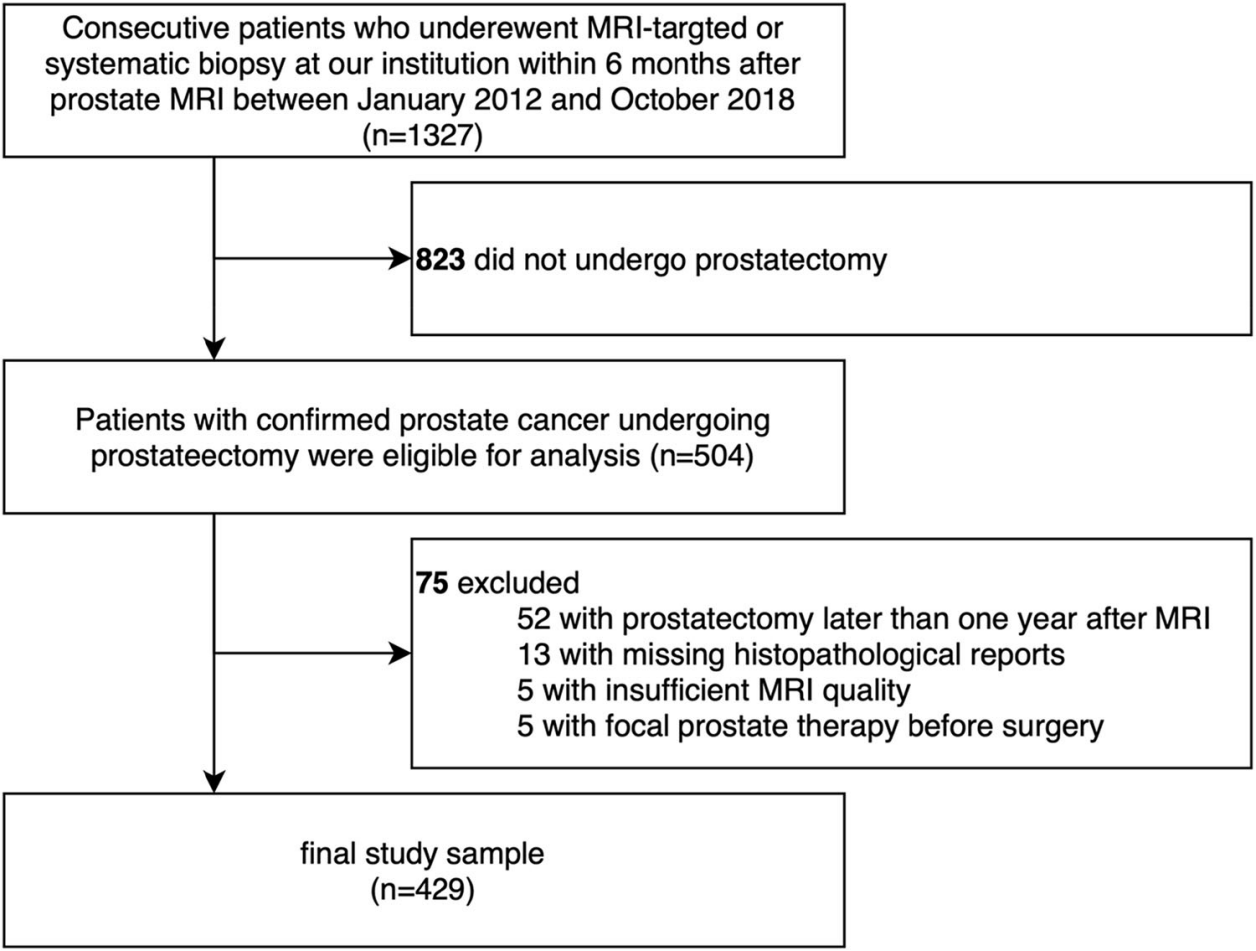
Study sample

Consecutive patients with clinically suspected PCa undergoing MRI-targeted and/or systematic prostate biopsy (if negative findings at prebiopsy MRI) at our institution within 6 months after prostate MRI between January 2012 and October 2018 were assessed for eligibility. A subset of this cohort was included in previous analyses that did not investigate EPE at MRI [16, 17]. Patients not undergoing prostatectomy were excluded from further analysis. Patients with prostatectomy more than one year after MRI, missing PSA values, MRI of insufficient quality, focal therapy prior to prostatectomy (e.g., brachytherapy, radiation, high-intensity focused ultrasound), and missing histopathological reports were also excluded (Fig. 1).

### MRI protocol and PI-RADS scoring

MRI was acquired according to the PI-RADS and ESUR technical recommendation guidelines and has been reported previously [16, 18, 19]. Imaging was performed on 1.5-T (Magnetom Avanto, Siemens Healthineers) and 3-T MRI scanners (Magnetom Skyra, Siemens Healthineers) using an MRI protocol comprising two-plane T2-weighted imaging (T2W), diffusion-weighted images, and a dynamic-contrast enhanced sequence, if available. All MR images were reviewed for sufficient image quality according to national diagnostic requirements for prostate MRI prior to analysis [20]. Under the supervision of expert radiologists (P.A. and T.P., each with > 10 years of experience), prostate lesions described by radiology faculty on primary reports were reviewed in consensus by two radiologists (C.A.H. and N.L.B., with 3–5 years of experience) using the PI-RADS v.2.1 criteria [19] and a rEPE grade was assigned [7]. rEPE grading followed the approach by Mehralivand et al.: grade 0, no suspicion for EPE; grade 1, lesion with either curvilinear contact length > 1.5 cm or capsular bulge; grade 2, both curvilinear contact length > 1.5 cm and capsular bulge; grade 3, frank EPE or invasion of adjacent anatomic structures. MRI-defined lesions were annotated and correlated with histopathologic diagnoses extracted from official pathology reports, following a study-specific hierarchical annotation protocol that accounts for multiple lesions (see Supplementary Materials). Whole-gland prostate volume (PV) was assessed using a semiautomated three-dimensional segmentation tool on T2W volumes [17].

### Prostate biopsy

All MRI/US fusion-guided transrectal targeted biopsies were performed by a team of urologists (each with > 10 years of experience) at our tertiary university center. First, targeted biopsy of the prostate with three cores per target lesion was performed as described previously [21]. Secondly, systematic biopsy was performed with 10 cores from apex, lateral mid-gland, base, ventral, and paraurethral region bilaterally, respectively. Systematic biopsy was only performed if pre-biopsy MRI was negative (PI-RADS 1–2). Specimens were examined and analyzed by experienced pathologists (including S.S.) following the International Society of Urological Pathology (ISUP) guidelines [22].

### Prostatectomy and evaluation of extraprostatic extension

Retropubic radical prostatectomy was performed in patients with histologically confirmed PCa. Based on informed decision-making, nerve preservation was attempted whenever possible, and intraoperative frozen section examination of the resection margins was performed. Once the resection margins were found to be free of tumor, an anastomosis was created between the bladder neck and the urethral stump, and the operation continued. Subsequently, the resected specimen underwent macroscopic and microscopic examination by specialized pathologists (including S.S.) at our institution.

Specifically, the specimen was measured, stained, and then cut for microscopic analysis, with resulting segments fixed in paraffin. The prostate, seminal vesicles, and vas deferens were measured, weighed, and marked with different colors on the preparation surface for subsequent microscopic analysis. This ensured clarity in identifying resection margins and their relationships. After processing, specimens were examined under a light microscope for Gleason score (ISUP grade group (GG)), TNM stage, and assessment of factors such as EPE, vascular invasion, perineural invasion, and seminal vesicle invasion.

### Outcomes and statistics

The primary outcome of this study was to identify disease-specific patient characteristics and imaging markers that enable the prediction of EPE at radical prostatectomy in patients with PCa. EPE was assessed in accordance with the guidelines established by the ISUP [23].

Mean and standard deviation (std) were utilized to describe continuous variables. Frequency and percentages were reported for nominal/categorical data. All imaging markers and disease-specific patient characteristics obtained were considered in the prediction of EPE, reporting the odds ratio (OR). Each marker was tested for individual prediction performance using a logit function with the Python statsmodels library. Afterwards, a multivariable test was performed to assess the independent prediction performance of the markers. A two-sided *p*-value < 0.05 was defined as statistically significant. Additionally, a clinically applicable decision tree classifier was developed on the available markers to define risk groups for EPE. We used an interpretable, rule-based decision tree classifier implemented in the scikit-learn library, based on the Classification and Regression Trees (CART) algorithm [24]. The algorithm constructs binary splits by recursively partitioning data to maximize Gini impurity reduction. Feature selection and split thresholds were determined automatically, retaining only the most informative variables. To ensure clinical interpretability and prevent overfitting, the maximum tree depth was limited to 3 nodes. Model development was performed independently of the logistic regression analyses. To assess generalizability and robustness, we performed 5-fold stratified cross-validation, reporting mean performance metrics with standard deviations. The final decision tree for clinical application was developed on the entire cohort.

Patient-based detection accuracy of EPE was reported by calculating sensitivity, specificity, negative predictive value (NPV), and positive predictive value (PPV) using bootstrapping over 1000 repetitions. Also, the clinical value of the developed decision tree was assessed using decision curve analysis. Statistical analyses were performed using Python (v.3.8.10) with the libraries scipy (v.1.5.4) and scikit-learn (v.0.23.2).

## Results

This study considered 1327 patients, of whom 429 were eligible for final analysis. The cohort’s mean age was 66.7 years (± 7.4), with a mean serum PSA value of 12.4 ng/mL (± 10.7; Table 1). Most patients (81.1%, *n* = 348) had an ISUP GG of 2 or higher at biopsy, and 99.1% (*n* = 425) had a PI-RADS score of 3 or higher. At a patient-level, rEPE grades 0, 1, 2, and 3 were assigned to 185 (43.1%), 112 (26.1%), 88 (20.5%), and 44 patients (10.3%), respectively. EPE was confirmed in 145 patients (33.8%) and ruled out in 284 (66.2%).

**Table 1:** Sample characteristics

Characteristic	Study sample *n* = 429 (*n*;%)
Mean age (y)	66.7
Mean PSA (ng/mL)	12.4
Mean PSAD (ng/mL^2^)	0.25
Size of index lesion at MRI (mm)	17.11
No. of patients with specified MRI index lesion	
None (PI-RADS 1–2)	4 (1)
PI-RADS 3	21 (5)
PI-RADS 4	152 (35)
PI-RADS 5	252 (59)
No. of MRI-index lesions with specified zonal distribution	
Peripheral zone	284 (66)
Transition zone	135 (31)
Multiple zones	10 (3)
No. of MRI-index lesions with specific regional distribution	
Dorsal	130 (30)
Ventral	116 (27)
Paramedian/medial	56 (13)
Lateral	126 (29)
Apex	152 (36)
Midgland	167 (39)
Based	109 (26)
Left	213 (51)
Right	203 (49)
No. of patients with EPE grade at MRI	
Grade 0	185 (43)
Grade 1	112 (26)
Grade 2	88 (21)
Grade 3	44 (10)
No. of patients with GG at biopsy	
Grade group 1	81 (19)
Grade group 2	113 (26)
Grade group 3	55 (13)
Grade group 4	131 (31)
Grade group 5	49 (11)
No. of patients with GG at prostatectomy	
Grade group 1	26 (6)
Grade group 2	184 (43)
Grade group 3	138 (32)
Grade group 4	31 (7)
Grade group 5	50 (12)
No. of patients with EPE at prostatectomy	145 (34)
No. of patients with local tumor staging at prostatectomy	
T1	1 (< 1)
T2	279 (65)
T2a	26 (6)
T2b	14 (3)
T2c	239 (56)
T3	144 (34)
T3a	96 (22)
T3b	48 (11)
T4	0 (0)
Not specified	5 (1)
No. of patients with vascular invasion	9 (2)
No. of patients with perineural invasion	53 (12)
No. of patients with seminal vesicle invasion	49 (11)

*EPE* extraprostatic extension grade, *ISUP GG* ISUP grade group, *PSA* prostate-specific antigen, *PSAD* prostate-specific antigen density at MRI, *PI-RADS* prostate imaging-reporting and data system score

### Association of imaging-derived markers and biopsy outcomes with EPE

**Fig. 2:**
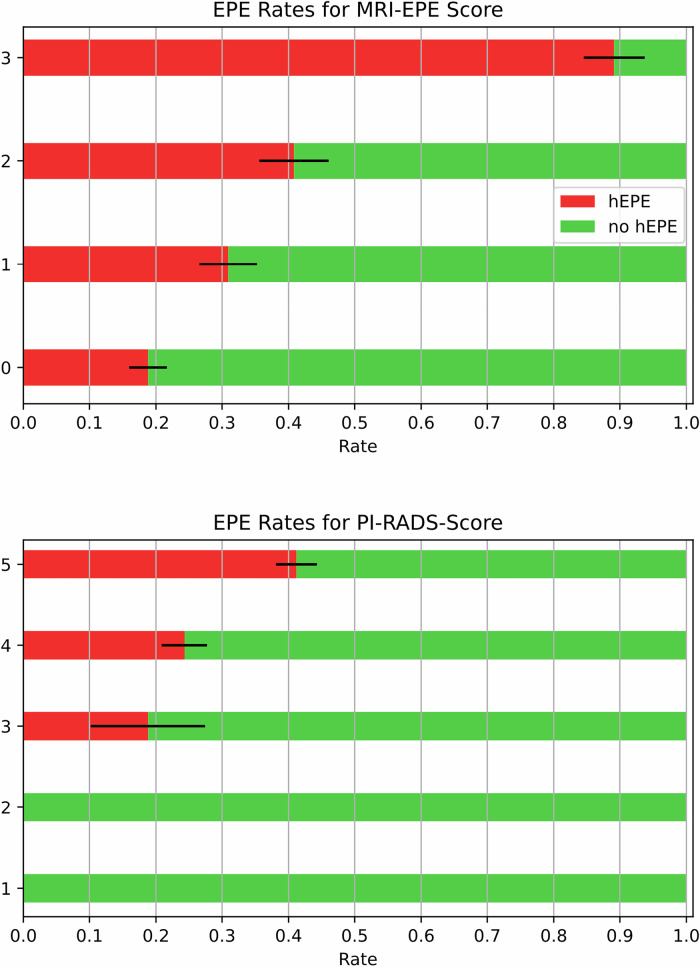
Association of PI-RADS score and EPE grade at MRI with histologically confirmed extraprostatic extension. Distribution of patients with histological extraprostatic extension (hEPE; red) according to the respective PI-RADS score and radiological EPE grade at MRI. The *x*-axis shows the respective score or grade assigned at MRI. The *y*-axis shows the percentage of patients with EPE

As the PI-RADS score and rEPE grade increased, so did the prevalence of EPE (Fig. 2). In patients with a PI-RADS score of 3, 19.1% showed EPE, while scores of 4 and 5 were associated with EPE rates of 24.3% and 41.3%. In comparison, EPE was observed in 19% of patients with an rEPE grade of 0, 31% with a grade of 1, 41% with a grade of 2, and 89% with a grade of 3.

Univariate logistic regression demonstrated associations between PI-RADS score (OR 2.07, *p* < 0.001), rEPE grade (OR 2.35, *p* < 0.001), GG at biopsy (OR 1.51, *p* < 0.001), MRI-acquired PSA density (PSAD) (OR 6.63, *p* < 0.001), PSA (OR 1.05, *p* < 0.001), and lesion size at MRI (OR 1.07, *p* < 0.001) with EPE (Table 2). On the contrary, rEPE grade (OR 2.64, *p* < 0.001) and GG at biopsy (OR 1.41, *p* < 0.001) remained the only statistically significant associations with EPE in multivariable logistic regression.

**Table 2:** Predictive power of imaging- and disease-specific markers for histologic extraprostatic extension

Marker	OR (95% CI)	*p*-value
Univariate logistic regression		
PI-RADS	2.07 (1.43–2.99)	< 0.001
rEPE	2.35 (1.89–2.93)	< 0.001
ISUP GG at biopsy	1.51 (1.29–1.78)	< 0.001
PSAD	6.63 (2.67–16.43)	< 0.001
Index lesion size at MRI	1.07 (1.04–1.1)	< 0.001
PSA	1.05 (1.02–1.07)	< 0.001
Age	1.01 (0.98–1.03)	0.62
Multivariate logistic regression		
PI-RADS	0.78 (0.49–1.25)	0.30
rEPE	2.64 (1.8–3.72)	< 0.001
ISUP GG at biopsy	1.41 (1.17–1.7)	< 0.001
PSAD	1.07 (0.11–10.79)	0.95
Index lesion size at MRI	0.98 (0.93–1.02)	0.28
PSA	1.05 (0.98–1.12)	0.19
Age	0.99 (0.96–1.03)	0.72

*rEPE* radiological extraprostatic extension grade, *ISUP GG* ISUP grade group, *PSA* prostate-specific antigen, *PSAD* prostate-specific antigen density at MRI, *PI-RADS* prostate imaging-reporting and data system score

The predictor with by far the highest PPV is an rEPE grade of 3. 39 of 44 (89%) patients with an rEPE grade of 3 had EPE, but this marker had a rather low sensitivity of 27% since most EPE cases had a grade < 3 (Supplemental Table 1). This finding highlights the importance of additional predictors in cases of moderate EPE risk. PSAD > 0.2 ng/mL^2^ had a PPV of 52%, and ISUP GG > 4 reached 55%. On the contrary, several markers demonstrated a high NPV for EPE. GG 1 PCa at biopsy, PI-RADS score < 4, rEPE grade of 0, and PSAD < 0.2 ng/mL^2^ demonstrated a high NPV of 85%, 84%, 81%, and 78%, respectively (Supplemental Table 1).

### Clinical utility and net benefit of the developed decision tree for EPE risk assessment

**Fig. 3:**
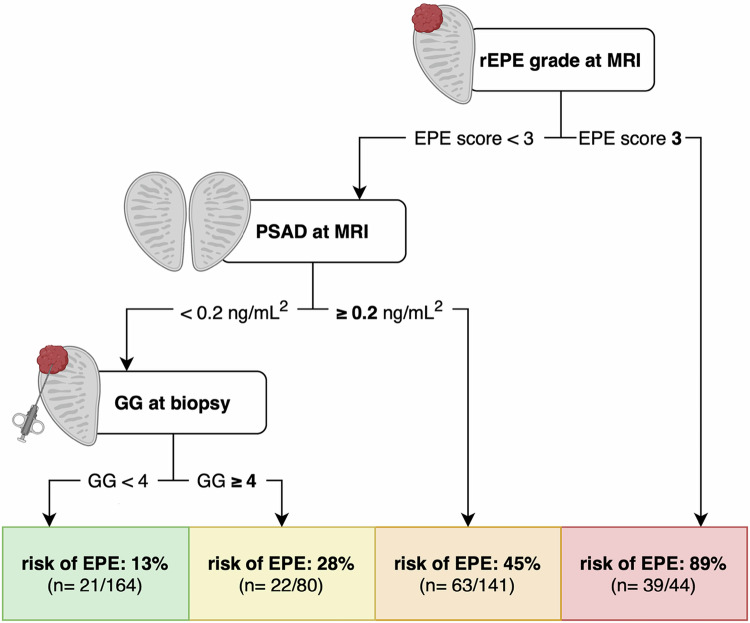
Clinical decision tool for assessing risk of histological extraprostatic extension. Risk for EPE based on radiological (r)EPE grade at MRI, prostate-specific antigen density (PSAD) at MRI, and grade group (GG) at biopsy. Note how 48% of the cohort can be reliably assigned to the low (13%) and high-risk (89%) groups, potentially allowing for more personalized treatment planning in these men

We developed a decision tree for EPE risk assessment fitted to the entire cohort to provide a clinically applicable tool. In the 5-fold cross-validation, the model demonstrated robust performance (AUC: 0.72 ± 0.04; accuracy: 0.74 ± 0.04). The final decision tree algorithm automatically selected three out of all available predictors as the most relevant markers to stratify EPE risk: rEPE grade, MRI-acquired PSAD, and GG at biopsy (Fig. 3 and Supplementary Materials). Following the developed decision tree, clinicians should first consider the reported rEPE grade, as men with a rEPE grade of 3 have a very high risk of EPE (high-risk group; 89% EPE risk). If the rEPE grade is ≤ 2, PSAD < 0.2 ng/mL^2^, and biopsy reveals a GG < 4 PCa, the risk of EPE is less than 13% (low-risk group). All patients outside of the high- and low-risk groups are regarded as the intermediate-risk group with an EPE risk of 28–45% (Fig. 3). Overall, the decision tree assigned 48% (208/429) of patients to the high-risk and low-risk groups, while 52% showed intermediate EPE risk.

The developed three-node strategy (rEPE score 3 or PSAD ≥ 0.2 or ISUP GG ≥ 4) and two-node strategy (rEPE score 3 or PSAD ≥ 0.2) provide the highest net benefit when clinicians accept a liberal threshold, meaning they would escalate treatment even if only about 1 in 20 patients truly has EPE (Supplementary Fig. 1). However, these strategies lose clinical utility when requiring that roughly every other patient flagged actually has EPE. By contrast, the most restrictive single-node strategy (EPE-Score 3 alone) starts with a lower net benefit but maintains positive clinical utility even at very stringent thresholds, remaining useful when clinicians demand that approximately 9 out of 10 patients flagged for EPE truly have it. This indicates that for patients and clinicians who want to minimize unnecessary treatment escalation, EPE-Score 3 alone is the most reliable marker, while for settings where missing EPE carries greater consequences and some overtreatment may be acceptable, the combined multi-node strategies offer superior clinical value.

## Discussion

This study investigated various markers for predicting EPE, focusing on the rEPE grade determined at MRI in a large retrospective sample. The findings highlight the rEPE grade’s central role in preoperative EPE risk assessment, alongside PI-RADS scoring, PSAD, and GG at biopsy. The prevalence of EPE in this large cohort, with 33.8% (145/429), was consistent with other investigations, although men in our sample showed higher rates of high-grade PCa (GG ≥ 3, 54.7% vs 29.1%–51.0%) [3–5, 7]. Nonetheless, the diagnostic performance of the investigated markers aligned with observations by Mehralivand et al and Park et al [4, 7]. rEPE at MRI proved to be the most accurate predictor of EPE, and multivariate analysis showed that rEPE grade and GG at biopsy were significant markers of EPE [4, 7]. However, our findings support previous observations that MRI-derived markers fail to demonstrate high sensitivity and specificity to function as exclusive markers in clinical decision-making regarding EPE risk [15]. Recent work has shown that combining MRI features with key clinical and biopsy variables can improve preoperative prediction of EPE compared with using either MRI or clinical parameters alone. In particular, an EPE risk model integrating standardized MRI assessment with factors such as PSA, PV, and GG at biopsy enabled more individualized (including side-specific) risk stratification that may better support nerve-sparing surgical planning [25]. These findings align with our approach of integrating rEPE with simple clinical markers to provide a pragmatic framework for preoperative EPE risk assessment.

In clinical practice, statistically significant markers often lack direct translation to practical decision-making tools. To address this gap, we developed a clinical decision tool incorporating three well-established and readily available markers using a binary decision approach for risk assessment. This tool enables reliable patient stratification into high-risk (rEPE ≥ 3) and low-risk (rEPE < 3 + PSAD < 0.2 ng/mL² + GG < 4 at biopsy) groups, with EPE risks of 89% and 13%, respectively. Given the parameters used, the tool is likely to be seamlessly integrated into clinical workflows without requiring additional image analysis. However, the decision tool classified only half of the patients (48%) into high- or low-risk groups, leaving 52% in an intermediate-risk category with a wide range of EPE risk. For these intermediate-risk patients, prostate-specific membrane antigen (PSMA) positron emission tomography/computed tomography (PET/CT) may provide additional precision in EPE risk prediction, as recent analyses have demonstrated good diagnostic performance when combining morphological information from MRI with PSMA expression patterns on PET/CT [26]. This tailored diagnostic approach could inform both surgeons and patients about surgical risks associated with tumor extent and help determine the need for radiation instead of surgery in men at high risk for EPE. Ultimately, this strategy contributes to more personalized cancer treatment decisions.

The study has some limitations. The retrospective, single-center study design harbors inherent selection bias and limits the generalizability of the findings. Our sample showed a high prevalence of GG ≥ 3 PCa compared to previous observations, potentially explained by different national treatment regimens employed at different institutions [3–5, 7]. Moreover, our rEPE demonstrated a very high PPV, which is likely explained by the higher EPE rate (33.8% vs 22.6% in Mehralivand et al [7]), resulting in a higher pre-test probability in the population, which shifts the PPV upward even if sensitivity and specificity remain similar. Also, two radiologists assigned rEPE grades in consensus under the supervision of two experts, likely reducing false-positive grade 3 assignments. Future studies should focus on validating the reproducibility and applicability of the proposed decision tool across multiple centers.

In conclusion, this study emphasizes the importance of MRI-derived markers for local staging of PCa using rEPE grade and PSAD. Specifically, our developed decision tool combining these two MRI-markers with tumor grade at biopsy enables the identification of men with high and low risk for EPE. Therefore, the proposed decision tool may enhance informed therapy decision-making and designing individualized treatment plans for PCa patients.

## Supplementary information

ELECTRONIC SUPPLEMENTARY MATERIAL

## Data Availability

Due to the sensitive nature of patient data involved in this study and the absence of explicit consent from study participants, study data cannot be made publicly accessible. For any specific inquiries or collaboration requests, please contact the corresponding author.

## Abbreviations

GG: ISUP grade group
ISUP: International Society of Urological Pathology
NPV: Negative predictive value
OR: Odds ratio
PI-RADS: Prostate imaging-reporting and data system
PPV: Positive predictive value
PSAD: Prostate-specific antigen density
PV: Prostate volume
rEPE: Radiological extraprostatic extension
T2W: T2-weighted imaging
