# Supplementary material for: Imaging-based clinical decision tree enables risk stratification of extraprostatic extension before radical prostatectomy in prostate cancer patients
Source: Insights Imaging. 2026 May 14;17:128. doi: 10.1186/s13244-026-02305-5 (PMC13172209; doi:10.1186/s13244-026-02305-5)
Supplement: Supplementary file 1 — ELECTRONIC SUPPLEMENTARY MATERIAL [file 13244_2026_2305_MOESM1_ESM.pdf]

Imaging-based clinical decision tree enables risk stratification of extraprostatic extension before radical prostatectomy in prostate cancer patients

ELECTRONIC SUPPLEMENTARY MATERIAL

Insights Imaging (2026) Hamm CA, Rüterhenke T, Baumgärtner GL, et al.

The index lesion selection followed this hierarchy:

1. **Single lesion:** The lesion was assessed for EPE grade and PI-RADS score, then matched with the corresponding lesion in the pathology report.
2. **Multiple lesions (≥2) with distinct EPE grades:** The lesion with the highest EPE grade was designated as the index lesion and matched with the corresponding pathology report finding.
3. **Multiple lesions (≥2) with identical highest EPE grades:** The lesion with the highest PI-RADS score was designated as the index lesion.
4. **Multiple lesions (≥2) with identical highest EPE grades and PI-RADS scores:** The lesion with the largest diameter was designated as the index lesion.

Supplemental Table 1. Diagnostic performance of imaging- and disease-specific markers and the decision tree for histologic extraprostatic extension.

Rule	Sensitivity (95% CI)	Specificity (95% CI)	PPV (95% CI)	NPV (95% CI)
**Individual marker thresholds**				
**rEPE grade >0**	75.86% [68.82%, 82.43%]	52.82% [46.99%, 58.70%]	45.08% [39.04%, 51.45%]	81.08% [75.14%, 86.41%]
**rEPE grade >1**	51.72% [43.38%, 59.60%]	79.93% [75.09%, 84.48%]	56.82% [48.12%, 64.96%]	76.43% [71.67%, 80.92%]
**rEPE grade >2**	26.90% [19.86%, 34.06%]	98.24% [96.52%, 99.65%]	88.64% [78.26%, 97.56%]	72.47% [67.86%, 76.73%]
**ISUP GG >1**	91.72% [86.96%, 95.95%]	24.30% [19.37%, 29.08%]	38.22% [33.33%, 43.28%]	85.19% [76.54%, 92.50%]
**ISUP GG >2**	68.97% [61.54%, 76.32%]	52.46% [46.89%, 58.04%]	42.55% [36.60%, 48.55%]	76.80% [71.00%, 82.29%]
**ISUP GG >3**	56.55% [48.34%, 63.91%]	65.49% [60.14%, 71.01%]	45.56% [38.25%, 52.63%]	74.70% [69.33%, 79.76%]
**ISUP GG >4**	18.62% [12.58%, 25.19%]	92.25% [89.08%, 95.24%]	55.10% [41.18%, 69.57%]	68.95% [64.28%, 73.35%]
**PSAD >0.10**	86.21% [80.30%, 91.31%]	23.59% [19.00%, 28.62%]	36.55% [31.52%, 41.67%]	77.01% [67.44%, 85.23%]
**PSAD >0.15**	71.03% [63.36%, 78.08%]	48.94% [43.15%, 54.77%]	41.53% [35.41%, 47.76%]	76.80% [70.37%, 82.44%]
**PSAD >0.20**	61.38% [53.15%, 68.84%]	70.77% [65.26%, 75.70%]	51.74% [43.78%, 58.95%]	78.21% [72.94%, 82.80%]
**PI-RADS score >1**	100.00% [100.00%, 100.00%]	0.35% [0.00%, 1.12%]	33.88% [29.74%, 38.32%]	100.00% [100.00%, 100.00%]
**PI-RADS score >2**	100.00% [100.00%, 100.00%]	1.41% [0.34%, 2.90%]	34.12% [29.88%, 38.55%]	100.00% [100.00%, 100.00%]
**PI-RADS score >3**	97.24% [94.29%, 99.37%]	7.39% [4.51%, 10.58%]	34.90% [30.58%, 39.46%]	84.00% [67.85%, 96.30%]
**PI-RADS score >4**	71.72% [64.29%, 78.67%]	47.89% [42.40%, 53.74%]	41.27% [35.43%, 47.20%]	76.84% [70.62%, 82.49%]
**Decision tree nodes**				
**Node 1. rEPE grade 3**	26.90% [19.86%, 34.06%]	98.24% [96.52%, 99.65%]	88.64% [78.26%, 97.56%]	72.47% [67.86%, 76.73%]
**Node 2. rEPE grade 3 and/or PSAD ≥0.2**	70.34% [62.41%, 77.70%]	70.77% [65.26%, 75.70%]	55.14% [47.75%, 62.21%]	82.38% [77.38%, 86.92%]
**Node 3. rEPE grade 3 and/or PSAD ≥ 0.2 and/or ISUP GG ≥4**	85.52% [79.22%, 90.91%]	50.35% [44.40%, 55.93%]	46.79% [40.58%, 52.79%]	87.20% [81.61%, 92.16%]

Note. – PPV=positive predictive value, NPV= negative predictive value, rEPE= radiological extraprostatic extension grade, ISUP GG= ISUP grade group at biopsy, PSAD= prostate-specific antigen density at MRI, PI-RADS= Prostate Imaging-Reporting and Data System score

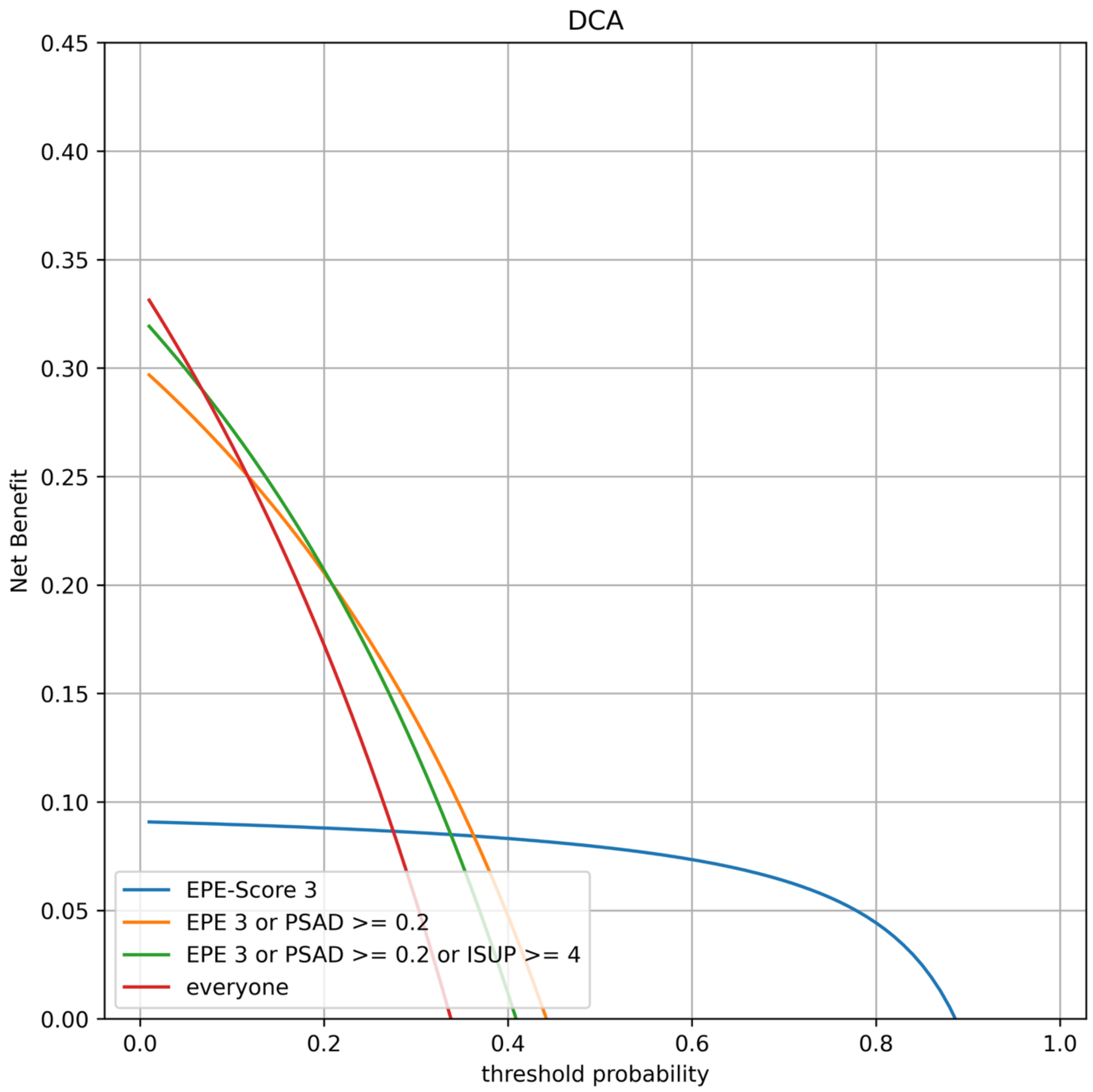
DCA
Net Benefit
threshold probability
0.45
0.40
0.35
0.30
0.25
0.20
0.15
0.10
0.05
0.00
0.0
0.2
0.4
0.6
0.8
1.0
EPE-Score 3
EPE 3 or PSAD >= 0.2
EPE 3 or PSAD >= 0.2 or ISUP >= 4
everyone

Supplemental Figure 1. Decision curve analysis comparing clinical utility of different extraprostatic extension risk stratification approaches. Decision curve analyses simulate two scenarios: in one, all men are considered to have histologic extraprostatic extension (hEPE) with more aggressive treatment planning (red), and in the other none would be (zero on the x-axis). Clinically useful strategies lie above these reference scenarios, and the threshold probability represents the minimum proportion of patients with true hEPE a clinician would require before escalating treatment. Based on the developed decision tree, three strategies were assessed: EPE score 3 alone (blue), EPE score 3 or prostate-specific antigen density (PSAD) ≥0.2 ng/mL^2 (orange), and EPE score 3 or PSAD ≥0.2 ng/mL^2 or ISUP grade group ≥4 (green). The multi-node strategies (orange and green) provide the highest net benefit at liberal thresholds (i.e., when clinicians would accept that as few as 1 in 20 flagged patients truly has hEPE), while EPE-Score 3 alone (blue) maintains clinical utility at stringent thresholds (i.e., requiring approximately 9 out of 10 flagged patients to truly have hEPE).
